# Reconstructing colonization dynamics to establish how human activities transformed island biodiversity

**DOI:** 10.1038/s41598-024-55180-9

**Published:** 2024-03-04

**Authors:** Sean Tomlinson, Mark V. Lomolino, Atholl Anderson, Jeremy J. Austin, Stuart C. Brown, Sean Haythorne, George L. W. Perry, Janet M. Wilmshurst, Jamie R. Wood, Damien A. Fordham

**Affiliations:** 1 Present Address: School of Biological Sciences, University of Adelaide, Adelaide, SA 5005 Australia; 2 College of Environmental Science and Forestry, State University of New York, Syracuse, NY 13210 USA; 3 School of Culture, History and Language, Australian National University, Canberra, ACT 0200 Australia; 4 Ngai Tahu Research Centre, University of Canterbury, Christchurch, 8140 New Zealand; 5 Centre of Excellence for Biosecurity Risk Analysis, University of Melbourne, Parkville, VIC 3010 Australia; 6 School of Environment, University of Auckland, Auckland, 1142 New Zealand; 7 Ecosystems & Conservation, Manaaki Whenua Landcare Research, Lincoln, 7640 New Zealand; 8 The Environment Institute, University of Adelaide, Adelaide, SA 5005 Australia; 9 Center for Macroecology, Evolution and Climate, Globe Institute, University of Copenhagen, Copenhagen, 1353 Denmark; 10 Center for Mountain Biodiversity, Globe Institute, University of Copenhagen, Copenhagen, 1353 Denmark; 11 Globe Institute, University of Copenhagen, Copenhagen, 1353 Denmark

**Keywords:** Island biodiversity loss, Human biogeography, Human migration, Process-based model, Pacific, Spatially explicit population model, Biodiversity, Biogeography, Ecological modelling, Macroecology, Palaeoecology

## Abstract

Drivers and dynamics of initial human migrations across individual islands and archipelagos are poorly understood, hampering assessments of subsequent modification of island biodiversity. We developed and tested a new statistical-simulation approach for reconstructing the pattern and pace of human migration across islands at high spatiotemporal resolutions. Using Polynesian colonisation of New Zealand as an example, we show that process-explicit models, informed by archaeological records and spatiotemporal reconstructions of past climates and environments, can provide new and important insights into the patterns and mechanisms of arrival and establishment of people on islands. We find that colonisation of New Zealand required there to have been a single founding population of approximately 500 people, arriving between 1233 and 1257 AD, settling multiple areas, and expanding rapidly over both North and South Islands. These verified spatiotemporal reconstructions of colonisation dynamics provide new opportunities to explore more extensively the potential ecological impacts of human colonisation on New Zealand’s native biota and ecosystems.

## Introduction

The emergence of hominids and their sequential dispersal away from an African evolutionary cradle has always been an intriguing topic for archaeologists, biogeographers, and conservation biologists^1^. However, key questions remain concerning the timing, rate and mechanisms influencing the rapid expansions of our species^2,3^ and the broader ecological consequences of human colonization on biodiversity^4,5^. This is particularly true for the colonisation of remote oceanic islands^6,7^, which are among the last areas on Earth to have been settled and transformed by people^8^.

While several potential pathways for the global expansion of modern humans have been proposed^3,9^, simulations of these colonisation dynamics have, to date, been done at relatively coarse spatiotemporal scales, often underpinned by an assumed positive correlation between net primary productivity and population growth in pre-agricultural societies^10^. Consequently, knowledge of drivers of human migration and their fine-scale dynamics are unclear, particularly for those that operated at spatial and temporal scales relevant to individual islands and archipelagos^11,12^.

Human arrival dates on many islands and archipelagos have been established archeologically with reasonable certainty^13^. While these dates have often been used to speculate on the role and impact of human activities on island biodiversity^8,14^, this has typically been done without considering the additional and important roles that founding population size and location, and rate and pace of expansion could have had on the spatiotemporal pattern of biodiversity. This oversight has not been intentional, but rather has occurred because of an absence of high-resolution reconstructions of human migrations across islands, which is difficult to establish, and remains heavily contested for most islands^15–17^.

Improving knowledge of the processes responsible for the transformation of native insular biotas following human arrival and expansion requires new methods that can reconstruct human colonization dynamics at spatiotemporal resolutions required for biodiversity assessments. These include assessments of the causal role of people in extinctions of island endemics, and resultant changes in the ecological function of islands across the Pacific Ocean^18^, Indian Ocean^19,20^, and the Caribbean^21^. New methods in macroecology that synthesize disparate evidence from archaeological records have the potential to reconstruct human events at spatiotemporal resolutions requisite for establishing human-mediated biodiversity change on islands^22^. However, their application to island systems has yet to be tested.

Part of the challenge with spatiotemporally reconstructing the dynamics of initial human migration across individual islands and archipelagos is that most remote islands were settled rapidly and relatively recently, when climates were similar to current conditions^8^. Consequently, these events cannot be reconstructed adequately in space and time using existing correlative techniques^23^, or climate proxies^9,10^. A potential solution could be to integrate archaeological information with spatially explicit population models (SEPM) that can reconstruct fine-scale dispersal and population dynamics using process-driven approaches and pattern-oriented methods^24^. Process-explicit modelling approaches simulate the dynamics of a biological system as explicit functions of the events that drive changes in that system^25^. When coupled with pattern-oriented modelling (POM) methods^26^, process-explicit models can establish chains of causality likely to be responsible for colonisation and extinction dynamics^27^, and resultant biodiversity change^28^. Critically, the approach has substantial potential for reconstructing rapid human expansion at relatively fine spatial scales, including those across oceanic islands and archipelagos during periods of climatic stasis^24^, providing vital information for disentangling human impacts on biodiversity^29^.

The Māori expansion across New Zealand provides an intriguing and insightful model system to demonstrate how the colonisation and subsequent expansion dynamics of humans across islands can be reconstructed using an approach that combines SEPMs^30^ with POM methods^26^. This is because there is a wealth of precisely dated archaeological evidence of Māori activity^31^, existing models of human population growth^32,33^, and eighteenth century estimates of Māori population size^34^. Just as importantly, there is an immediate need for a more detailed understanding of the pattern and pace of Māori migration across New Zealand to better understand the role past human activities had in the dynamics and extinctions of New Zealand’s native biotas. This is because current assessments of biodiversity change following the peopling of New Zealand have rarely considered the consequences of founding location, or rate and pattern of human expansion across the archipelago^33,35^.

The East Polynesian expansion in the Pacific Ocean was the final phase of global human settlement^12^. It included the colonisation of the New Zealand archipelago by Polynesians known subsequently as Māori. Archaeological evidence suggests an expansion that was so rapid as to appear highly synchronous across the entire archipelago^15,36^. Consequently, there remains little consensus on the location of first arrival, migration routes and whether colonisation resulted from a series of small founding populations or a single, concerted migration^12,15,37^: information urgently needed to better understand the human dimension of biodiversity change in New Zealand, including loss of its megafauna.

These colonization dynamics cannot be resolved using existing human-migration models, partly because they rely on climatic change (and derived changes in net primary productivity) as the principal drivers of colonisation and expansion^9,10^. However, climatic conditions in New Zealand in the centuries immediately prior to and during initial colonisation (1200–1300 AD) were relatively stable^38^, providing no insights into the establishment and spread of people across New Zealand, nor their subsequent spatiotemporal impacts on native biotas. Moreover, Polynesian colonists were not entirely dependent on hunting and gathering^32,39^, for which net primary productivity is a proxy^10^. It is likely, however, that these limitations can be overcome using process-explicit models, archaeological records and climate and environmental data^22^.

The process-explicit, pattern-oriented modelling framework that we develop and test here, simulates the colonization and establishment of people in New Zealand, providing great potential for understanding how Māori transformed island biodiversity. More generally, it can be used to reconstruct the initial waves of human colonisation across other remote, large islands and archipelagos for which similar data are available, potentially providing novel insights into fine-resolution drivers of biodiversity change following human arrival.

## Methods

**Figure 1:**
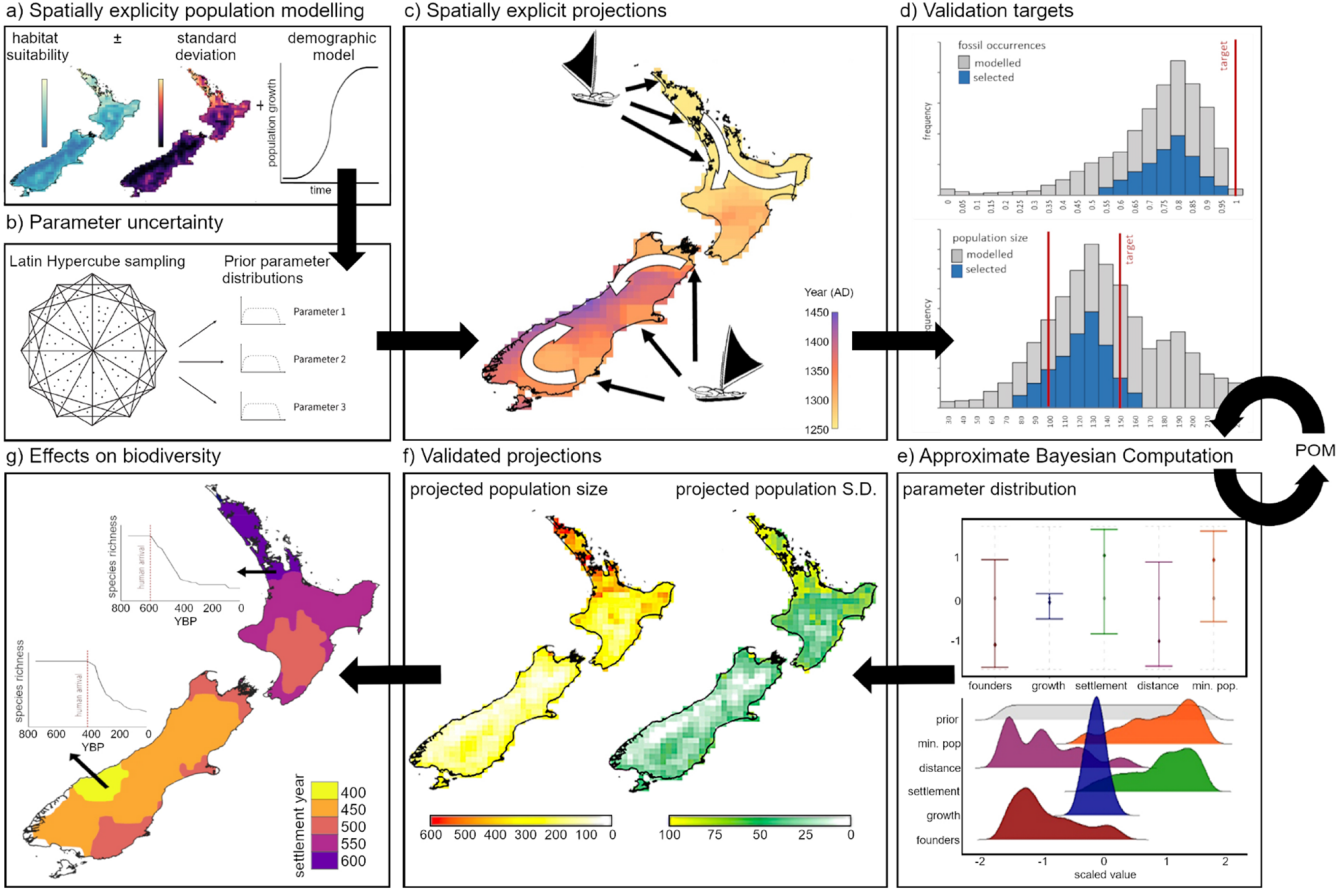
Reconstructing Māori colonisation dynamics using spatially explicit population modelling (SEPM) with pattern-oriented modelling (POM). (**a**) Spatiotemporal estimates of Māori relative density were combined with demographic models to simulate colonisation, population growth and geographic expansion. (**b**) To account for parameter uncertainty, thousands of potential models were generated using Latin hypercube sampling, and (**c**) each model was simulated, providing a plausible spatiotemporal projection of arrival time, range expansion and population abundance. (**d**) Model projections were validated against inferences from archaeological archives, and (**e**) the most accurate projections were selected using Approximate Bayesian Computation. The frequency distribution of parameters in these best models were compared to their frequency distribution for all models, and if they differed the processes was repeated. (**f**) Once the parameters converged, the best models were used to project population abundance in space and time, which can be used to (**g**) establish the role of human activities on changes in island biodiversity, including shifts in species distributions, changes in species richness and shifts in ecosystem structure and function. All maps were generated in the R statistical environment using the ‘*raster*’ package v3.4-5^82^ and the ‘ggplot2’ package v3.4.4^83^.

Our new statistical-simulation approach for reconstructing human colonisation dynamics on islands at high spatiotemporal resolutions integrates archaeological data with population growth and dispersal models to produce dynamic simulations of changing populations, distributions and migration routes of people at fine spatiotemporal resolutions (Fig. 1). It leverages off coarser scale models of first human migration across continents^40,41^, and recent extensive use of process-explicit models for reconstructing past biodiversity change^27,29,42^. Archaeological records matched with climate and environmental data are used to reconstruct environment and climatic suitability for human occupancy on islands, and relative density patterns at spatial resolutions that capture local orographic influences (Supplementary Fig. S1). This information is integrated into spatially explicit population models (SEPMs) that simulate population growth and dispersal dynamics. Uncertainty is captured directly in simulations by varying model parameters (demographic, dispersal, suitability, and density parameters), producing thousands of conceivable models of human arrival and establishment (Fig. 1). Pattern-oriented modelling (POM) is used to optimise parameter values using inferences of demographic change from archaeological and historical records. Models that validate well are used to reconstruct human colonisation and establishment, identify causative processes responsible for spatiotemporal patterns of abundance, generating information needed to determine past influences of people on biodiversity^27^. Resultant conclusions can be tested using counterfactual scenarios that modify the effects of these parameters^43^.

Below we describe the application of this approach to the colonization of New Zealand by Māori. Models are coded in Program R version 4.0.4 R Core^44^ and are described in detail in the Supporting Methods, and example simulations which are available here: https://figshare.com/s/02c292e2386633546e2e.

### Modelling Māori relative population density

**Figure 2:**
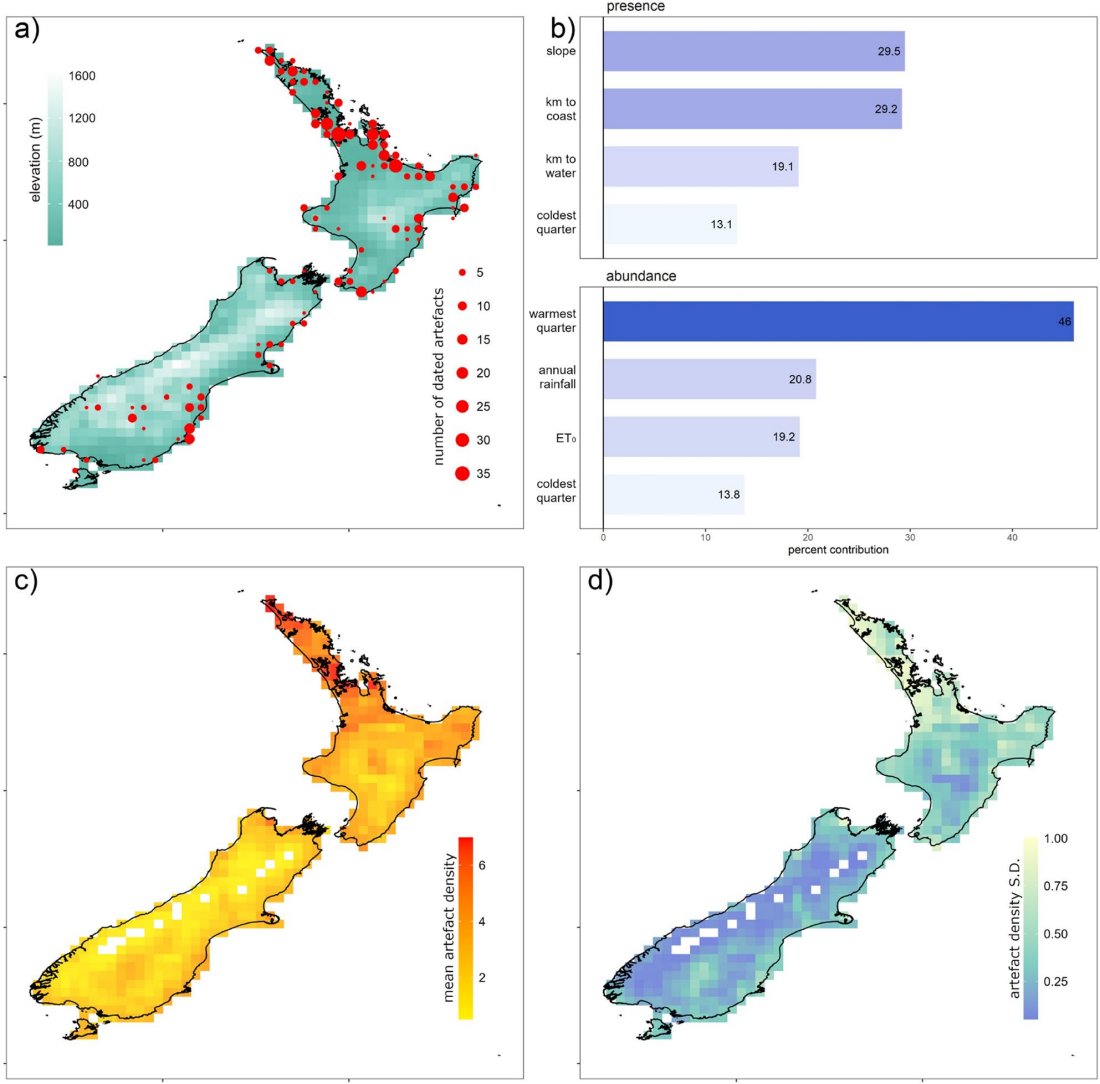
Reconstructing relative abundance of people using archaeological data. (**a**) ^14^C-dated archaeological samples across New Zealand during the colonisation period (1000–1650 C.E.) mapped at a 0.25° resolution. Lighter cells represent higher elevations. (**b**) Effect sizes for variables contributing to the probability of presence and the relative abundance of human samples (proxies for occurrence and abundance of people) across the period of colonisation in New Zealand (estimated using a boosted regression tree). (**c**) Map of projected mean relative density of human samples and (**d**) its standard deviation. Variables in (**b**) are the area of each grid cell steeper than 20° (slope), the distance to the coast (km to coast), the distance to navigable water (km to water), the average temperatures in the coldest quarter (coldest quarter) and the warmest quarter (warmest quarter) of the year, annual rainfall, and annual evapotranspiration (ET_0_). All maps were generated in the R statistical environment using the ‘*raster*’ package v3.4-5^82^ and the ‘ggplot2’ package v3.4.4^83^.

Spatial models of relative density of Māori populations prior to European first contact (conventionally 1769 C.E.) were constructed using the density of archaeological finds as a proxy for human density^40^. Specifically, we trained boosted regression tree models (BRT^45^) using radiocarbon (^14^C) dated Māori archaeological records sourced from The University of Waikato’s New Zealand Radiocarbon database (Fig. 2). These dated records of Māori occupation are a spatiotemporally representative subset, constrained to the colonisation period between 1000 and 1650 C.E., of the entire archaeological record of Māori in New Zealand, which has been digitised by the New Zealand Archaeological Association (Supplementary Fig. S1). In accordance with Māori data sovereignty principles, we did not use DNA or genomic data, nor traditional knowledge in model development^46^. We also ensured that there were no issues of Māori intellectual ownership, of mātauranga Māori or of any traditional beliefs with the data and its uses.

We intersected these archaeological records with paleoclimate data generated using PaleoView v1.5.1^47^, and geomorphometric data. PaleoView provides access to climate reconstructions that accurately reproduce major climatic features associated with the most recent deglaciation event, and predict present-day patterns of climate conditions (including for New Zealand) with verified hindcast skill^47^. To capture important orthographic elements in New Zealand’s climate, these anomalies were downscaled to a 0.30° × 0.30° grid cell resolution using a change factor method^48^.

We used a decomposed hurdle approach for BRT models^49^. This allowed the occurrence and abundance of ^14^C data to be trained on different environmental factors^49,50^ (Supplementary Fig. S2). This process also addressed zero inflation resulting from spatial absence of archaeological finds, or from a lack of ^14^C dated samples at some archaeological sites. The BRT model was used to project mean abundance of samples and its standard deviation across New Zealand at a grid cell resolution of 0.25° × 0.25° (Fig. 2). The BRT model and its validation are described in detail in the Supporting Methods.

### Spatially explicit population model (SEPM)

To reconstruct the likely colonisation dynamics of Māori from 1230 to 1850 AD, spatial projections of potential relative population abundance and its standard deviation (described above) were coupled with population growth and a dispersal models. This was done using a lattice-based SEPM framework^24^ that models range expansion annually as a function of population size (Supplementary Fig. S3) and habitable neighbourhoods (Supplementary Fig. S4). To do this for Māori, we used an exponential population growth model^33^, and colonised neighbourhoods sequentially. Neighbourhoods with the highest potential relative abundances (those in the most suitable areas for settlement) were colonised first. To do this, grid cells were grouped into spatial neighbourhoods using plausible foraging radii. This allowed dispersal and local population growth of Māori across New Zealand to be simulated as the total population grew (Supplementary Fig. S4), with local population growth rates being dependent upon the suitability of the landscape to Māori (see “Supplementary Methods”).

To account for potentially wide parameter uncertainty, we generated 25,000 simulations. We did this by varying five demographic and settlement and expansion parameters in the SEPM across large but plausible ranges (Table 1) using a robust coverage of multi-dimensional parameter space^51^. Variable parameters were time of arrival, founding population, population growth rate, neighbourhood size, and foraging distance. The SEPM was built using the ‘*poems*’ version 1.0.1 Program R package^52^. A detailed description of the mechanics of the model is provided in the Supporting Methods.

**Table 1:** Parameter values used in the process-explicit model (POM) of Māori colonisation and expansion.

*poems* parameter	Description	Type	Values	Credible interval
Spatiotemporal template				
simulation_start_year	The year at which all simulations were initiated	fixed	850 AD	NA
time steps	Simulation years	fixed	1101	NA
lattice	Number of 0.25° grid-cells	fixed	431	NA
Demographic parameters				
human_founding_population	The number of Polynesian colonists that first arrived in New Zealand	variable	200–600	435–582^a^
human_growth_rate	The rate at which the Māori population increased following arrival in New Zealand	variable	1.005–1.050	1.010–1.011^a^
Settlement and expansion parameters				
human_colonisation_time	The year (AD) in which Polynesians colonised New Zealand	variable	1230–1314	1233–1257^b, c^
human_foraging_distance	The radius (km) of the foraging range of each Māori settlement	variable	30–70	63–68
human_min_neighbourhood	The minimum number of people required to seed a new Māori settlement	variable	20–80	23–36

Fixed values were consistent across all simulations, while variable parameters were allowed to vary randomly across the entire parameter space (Type). Fixed and prior values for parameters are provided (Values). Posterior values for variable parameters according to POM validation are shown (Credible Interval). Superscript letters indicate published sources for credible intervals; a = ^33^; b = ^63^; c = ^12^.

### Pattern oriented model validation

POM methods are being used with increasing frequency to optimise parameters in SEPMs^29,52,53^. This is being done by comparing model simulations with independent validation targets and selecting models that have the mechanisms to most closely replicate these targets^26^, often using Approximate Bayesian Computation (ABC^54^).

Model simulations of Māori arrival and expansion in New Zealand were assessed against two targets: (i) Spatiotemporal occurrence, measured as modelled presence in grid cells at a time and place where ^14^C-dated archaeological evidence indicated that that the grid cells should have been occupied; and (ii) Population size (and its uncertainty) at the time of European arrival, estimated at 100,000 to 150,000 people across the archipelago in 1769 C.E.^34^. The best 1% of simulations were selected using the rejection algorithm in the ‘*abc*’ package^55^. The parameter ranges identified by ABC as most accurately matching the validation targets were used to build additional simulation models (n = 25,000), using the posteriors of previous model runs as informed priors^25^. This POM process was stopped when Bayes factors indicated that the selected posteriors no longer differed from the informed priors^56^. Posterior predictive checks were used to determine whether the posterior distributions generated strong resemblance between the simulation results and observed data^56^. See the Supporting Methods for further details.

### Model output and sensitivity analysis

To reconstruct human colonization patterns, we calculated credible intervals for model parameters from the ‘best’ 1% of optimised simulations and then generated multi-model averaged projections of time and location of first arrival of Māori in New Zealand, founding population size, and population growth and migration through space and time. Projections were weighted by ABC model weights^27^.

We determined the sensitivity of the results to parameter uncertainty and two common model-based structural assumptions^57^: the form of the population growth model; and the number of founding events. To do this, 25,000 simulations were generated using a robust coverage of the posterior parameter space identified by POM. Parameter uncertainty was tested using a global sensitivity analysis^58^. Specifically, we built Bayesian linear models using the ‘*rstanarm*’ R package^59^ to assess whether all five variable demographic parameters were needed to reconstruct inferences of Polynesian colonisation from the archaeological record. Parameters were regressed against the Euclidean distance from an idealised model. Models were constructed with uniform priors, each with 25,000 samples. We checked model convergence using Gelman-Rubin statistics (where values less than or equal to 1.1 were considered acceptable), tested effective sample size, and visually examined trace-plots.

We also assessed the effects of model structure on the performance of our simulations, identifying two aspects of our models to validate: the function of human population growth, and the number of founding events. Patterns of human population growth were assessed by altering the human growth function so that it followed a logistic, rather than exponential, function^32^. The effects of founding events were explored by simulating multiple arrivals rather than a single fleet. Where multiple founding events were simulated, founding populations were spread over multiple time steps. Model outputs were compared to simulations without these structural changes. See Supporting Information for further details.

## Results

### Māori relative population density

The likelihood of occurrence of ^14^C-dated archaeological samples was higher in areas with fewer steep slopes (i.e. > 20°) and those closer to navigable waters (Fig. 2; Supplementary Fig. S2). Higher relative densities of Māori, based on greater numbers of ^14^C-dated Māori archaeological records, were projected to occur in areas where average temperatures during the warmest three months of the year exceeded 18 °C, temperatures in the coldest three months exceeded 10 °C, where evapotranspiration (and thus horticultural productivity) was high, and where rainfall was limited, preventing water logging of crops (Fig. 2; Supplementary Fig. S2). The reconstructed pattern of Māori relative density (based on ^14^C data) aligned closely with the distribution of all archaeological material, with 90% of all archaeological sites having > 0.75 likelihood of Māori occupancy (Supplementary Fig. S5).

### Colonisation dynamics

**Figure 3:**
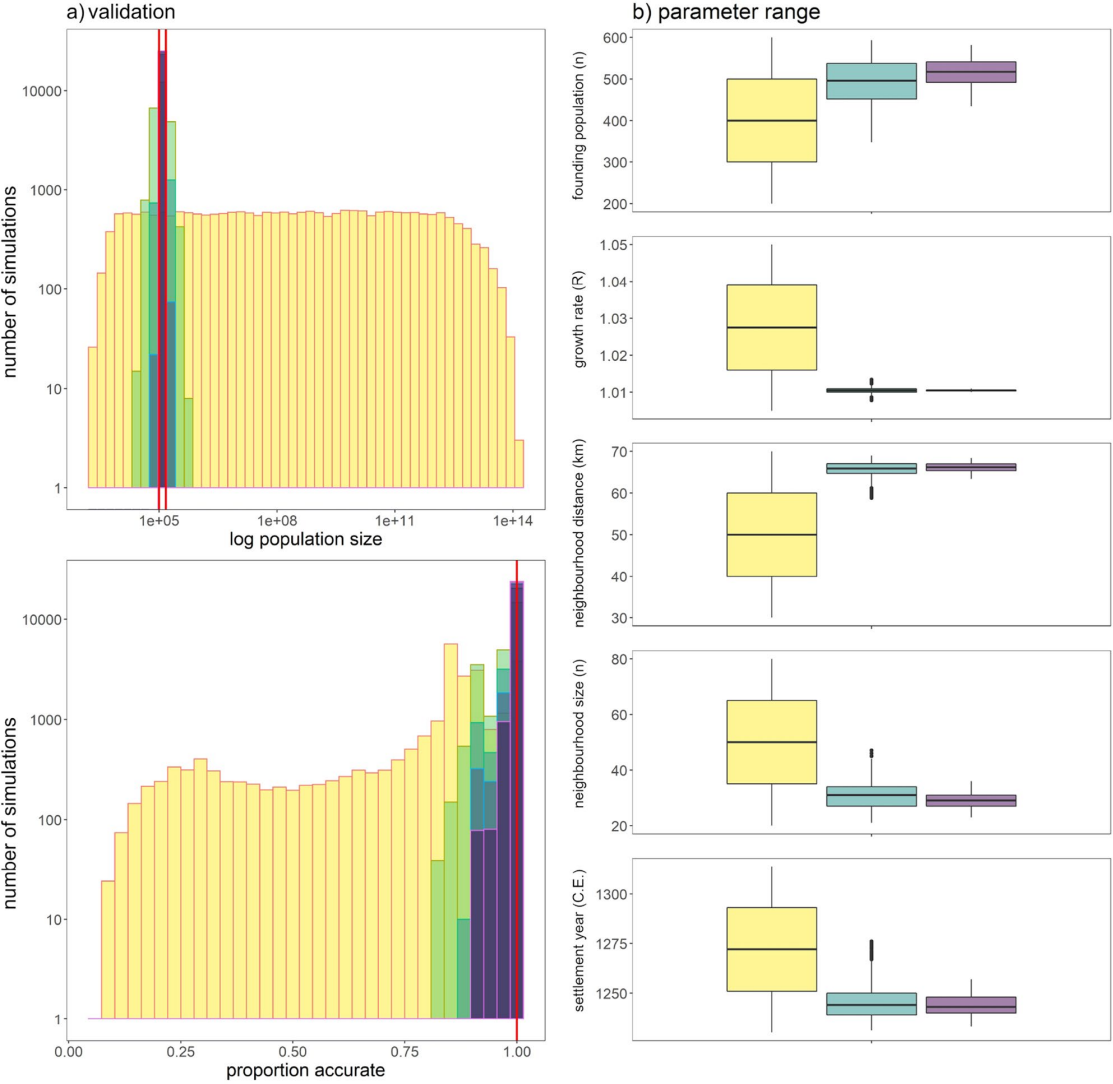
Estimates of settlement and colonisation of New Zealand by Māori using pattern-oriented modelling (POM). (**a**) Histograms show differences between simulated frequencies and observed targets for three iterations of POM, refined using Approximate Bayesian Computation (ABC). Top histogram shows results for population size at time of first European contact (plotted on the log scale). Bottom histogram shows the proportion of archaeological records accurately modelled in space and time. Red solid lines indicate validation targets. (**b**) Box plots show ranges for model parameters resulting from reiterative ABC resampling. In (**a**,**b**), yellow represents the first iteration, green indicates the second iteration, and purple indicates the third iteration.

Reconstructing POM validation targets for human colonisation of New Zealand required a constrained set of ecological parameters. These were a founding population size of 517 (range: 435–582), a colonisation year of 1244 C.E. (1233–1257), a minimum community size of 28 individuals (23–36), a neighbourhood radius of 66 km (63–68), and a population growth rate of 1.010 per annum (1.010–1.011) (Table 1, Fig. 3). While the first iteration of POM (with broad uninform priors) resulted in selected SEPMs that replicated colonisation patterns reasonably well (Fig. 3), the second and third iterations of POM did better, placing Māori colonists at nearly all known settlements prior to the earliest radiocarbon dated evidence of their presence there (Fig. 3). The best SEPMs of the third iteration yielded estimates of population size in 1769 [119,900 (88,750–159,197)], which most closely matched the target (Fig. 3; Supplementary Fig. S6).

**Figure 4:**
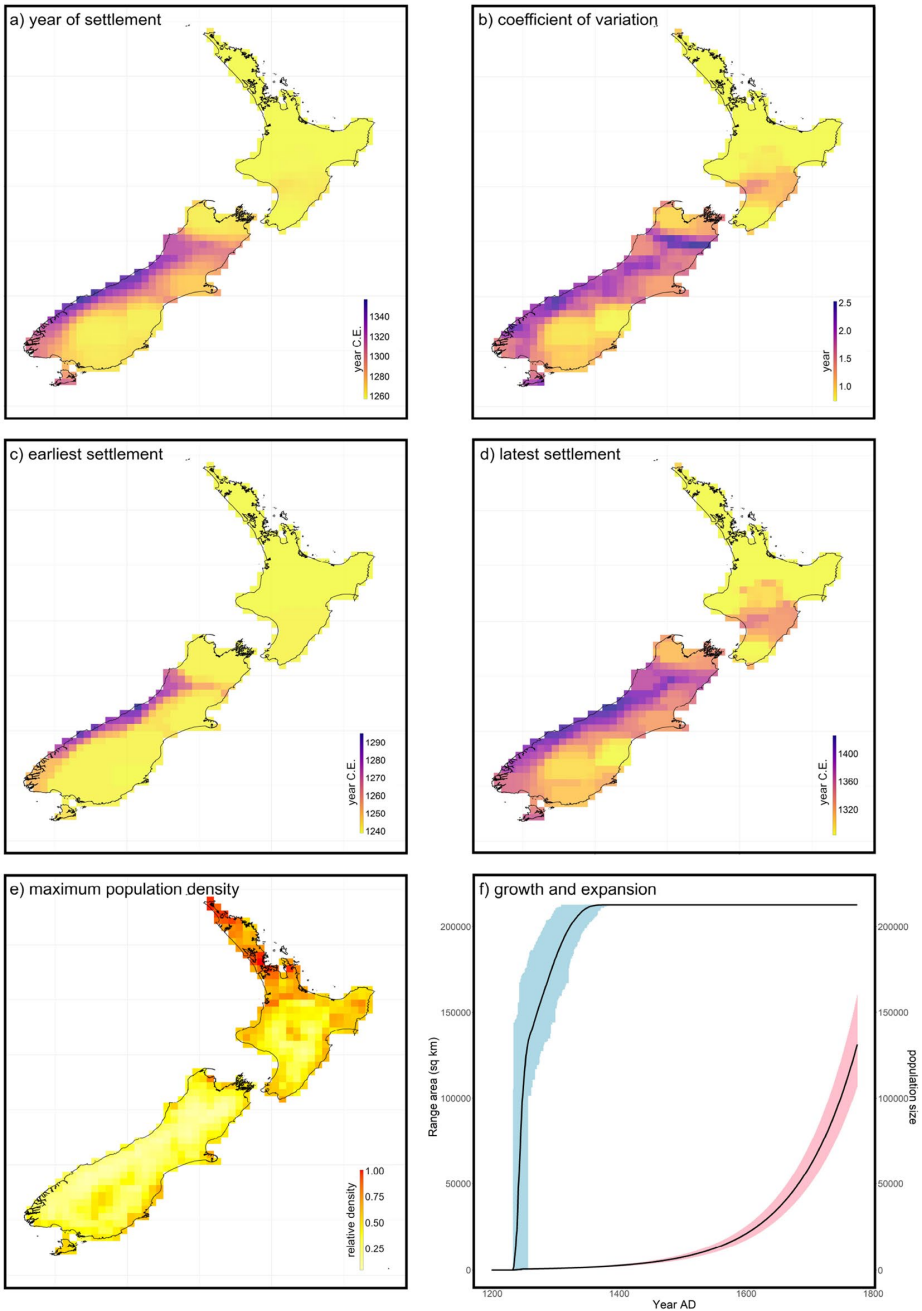
Island colonisation dynamics. Spatial estimates of (**a**) mean colonisation year and (**b**) its coefficient of variation, (**c**) earliest and (**d**) latest estimated year of colonisation, and (**e**) maximum population density. Estimates are multi-model ensemble average based on the 250 spatially explicit population models that best reconciled validations targets. (**f**) Estimated rate of range expansion (blue) and population growth (pink), with weighted multi-model averaged value shown in black. All maps were generated in the R statistical environment using the ‘*raster*’ package v3.4-5^82^ and the ‘ggplot2’ package v3.4.4^83^.

The best 1% of SEPMs (from the third POM iteration) consistently simulated the North Island being colonised prior to the South Island (Fig. 4). They simulated the colonisation of New Zealand as occurring rapidly, with the entirety of habitable regions colonised by approximately 1400 C.E.; i.e., within 200 years of arrival (Fig. 4; Supplementary Animation S1). On the South Island, present day Otago, Canterbury, Marlborough and Nelson were projected to have been settled as early as the mid-1200s C.E. in some selected simulations (Fig. 4; Supplementary Fig. S1). While small differences in settlement and dispersal parameters between selected simulations caused some variation in reconstructions of occupancy and abundance, there was substantial spatiotemporal agreement between the best 1% of simulations for the pattern of Māori establishment of New Zealand (Supplementary Animation S2). Based on the multi-model average of selected models, approximately 63% of the Māori population lived in areas of present-day Northland, Auckland, Waikato, Taranaki and Bay of Plenty during the colonising period, a finding consistent with earlier suggestions that these regions harboured the largest Māori populations^32,60^. Areas of greatest population density occurred across the North Island, especially in present day Northland, Auckland, Bay of Plenty and Gisborne, and in Marlborough and Canterbury on the South Island (Fig. 4; Supplementary Fig. S1).

The global sensitivity analysis showed that all variable demographic parameters strongly influenced the capacity of SEPMs to reconstruct inferences of Polynesian colonisation from the archaeological record. Our projections of Māori arrival and expansion in New Zealand were most sensitive to human population growth rate and number of people required to found new communities, and least sensitive to the year of Polynesian colonisation (Supplementary Fig. S7). While model projections of spatiotemporal patterns of human abundance were not sensitive to the structural population growth function (i.e., logistic versus exponential; Supplementary Fig. S8), the number of founding events substantially altered the pattern and timing of colonisation, and these differences were magnified with increasing numbers of founding events (Supplementary Fig. S9). Total population size in the year 1769 C.E. was sensitive to both the type of population growth function and number of founding events (Supplementary Figs. S8 and S9). The implications of these observations are discussed below.

## Discussion

Given the integral role that human population growth and expansion has had on biodiversity declines during the Holocene^5,6^, understanding how humans colonised different islands and archipelagos in response to their unique environments is key to understanding the ecological consequences of these events^61,62^, including globally-significant declines in biodiversity^8^. However, absence of high-resolution reconstructions of patterns and paces of human migrations across islands continues to hinder the extent to which islands can be used as replicated model systems to establish processes of human transformation of biodiversity. We show that process-explicit models that are informed by archaeological records, and spatiotemporal reconstructions of past climates and environments, can provide new and important insights into the patterns and mechanisms of colonisation and establishment of people on islands, generating spatiotemporal reconstructions of human abundance at resolutions needed for biodiversity assessments.

Our SEPM projections of the arrival and expansion of Māori in New Zealand closely reconciled inferences of demographic and distributional change from the archaeological record, and more recent historical observations, revealing the importance of topography, proximity to navigable water bodies, and the geography of climatic conditions and habitats on colonisation dynamics. While these drivers have been identified as important in other studies of human biogeography^1,41^, our results provide a more processed-based understanding of their causality for Māori. Importantly, these verified simulations provide new opportunities to explore more extensively the potential ecological impacts of human colonisation on New Zealand’s native biota and ecosystems in space and time^35,63^, including the roles people have had on species distributions and changes in species richness and ecological function. More generally, the framework developed for reconstructing the colonization of New Zealand by Māori, is directly transferrable to other islands and archipelagos, where climate and archaeological records are available, and their access and use is both ethical and equitable^46^.

### Spatially explicit Insights on Māori colonisation

Polynesian expansion across the Pacific has been hypothesised to have resulted from carefully planned voyages^64^, however, there are alternative theories^15,65^. While our modelling indicates that Polynesian colonisation across New Zealand was highly synchronous, it does not imply planned migration because the prevailing winds during the colonisation period were highly favourable to reaching New Zealand from East Polynesia^66^. In either event, our simulations consistently resulted in early settlements arising nearly simultaneously in multiple locations, probably connected by coastal navigation routes. Parameter values in our models, chosen through pattern-oriented methods, are highly congruent with established estimates, including timing of arrival in New Zealand^12,63^, number of colonisers^15,37^, and spatial variation in population growth rates^32^. The areas projected by our models as the most likely sites of Māori first settlement also encompass sites with the oldest archaeological evidence of Māori presence, such as Wairau Bar, Houhora and Tairua^12,67^.

Simulation models are sensitive to structural assumptions that constrain them to operate in specific pre-determined ways. Two important structural assumptions in the Māori SEPM were the number of founding events and the type of population growth function. A global sensitivity analysis showed that increasing the number of independent founding events above one substantially alters the projected colonisation dynamics, resulting in a poorer match between model simulations and inferences of demographic change from the archaeological record. This suggests that New Zealand was likely to have been founded by a single colonisation event. However, this result must be viewed cautiously since other demographic parameters in the SEPMs with founding events greater than one were not optimised using POM approaches^25^. Nevertheless, our results show that a very high (and perhaps unrealistic) population growth rate would be needed to reproduce the archaeological record under a scenario of multiple founding events.

Both exponential and logistic functions have been used to model pre-European Māori population growth rates^32,68^. Our sensitivity analysis showed that while spatiotemporal patterns of human abundance were not sensitive to the choice of growth function, exponential population growth provided a closer fit to validation data. However, neither function, when applied to our modelling architecture, reconstructed a plateau in population growth at ~ 1500 AD, as has been inferred from the archaeological record^32^, perhaps indicating that Māori population dynamics were more complex than what was captured in our simulations. This could be because our model did not simulate complex spatiotemporal population dynamics, including boom-bust dynamics driven by the overhunting of large animals^69,70^.

An element of the Māori colonisation of New Zealand that we could not replicate was the putative abandonment of the South Island following the extinction of the moa, which has been inferred from the fossil and archaeological records^15^. Some authors have suggested that the South Island was never densely populated by Māori^71^, as indicated by our SEPM, and that sparse populations persisted following the depletion of wild food resources such as moa^32^. However, this runs contrary to the prevailing view that the South Island initially harboured large Māori populations who then shifted to the North Island when wild food sources were depleted^15^. It is likely, that the accuracy and interpretation of our estimates of the colonisation dynamics of Māori across New Zealand will improve with more extensive ^14^C-dating of archaeological material, more precise estimates of change in population size prior to European arrival, and higher-resolution paleoclimatic simulations.

### Ecological implications of rapid colonisation

The arrival and spread of humans across the world’s islands had substantial ecological consequences^7^, and the Polynesian colonisation of New Zealand was no different. The colonisation of New Zealand resulted in widespread deforestation^35^, and serious faunal population declines or wholesale extinctions^33,63^. However, until now, the timing, rate and magnitude of these anthropogenic impacts have been difficult to resolve because of the absence of a detailed spatiotemporal understanding of how Māori expanded across the archipelago.

Our new macroecological modelling approach for reconstructing the peopling of islands shows strong spatiotemporal variation in colonisation patterns of New Zealand and subsequent densities of people. We project that colonisation happened more rapidly on the North Island, spreading from the northwest of the island to the southeast. On the South Island the colonisation and spread of people is likely to have happened more slowly, spreading from the east coast of the island to the west. Given that human density and environmental change are strongly correlated at local-to-regional scales^4^, this fresh perspective on Māori colonisation dynamics is likely to provide important new insights into the ecological impacts of this rapid migration of humans across New Zealand.

The role of human colonisation dynamics on extinctions of New Zealand avifauna (and other fauna) is poorly understood^72,73^. Of the 131 known endemic species present in New Zealand at the time of Polynesian settlement, at least 40 became extinct prior to European arrival^14,73^. While these population declines and extinctions have been attributed to human impacts, their timings and geographic patterns have been difficult to discern even for well-studied birds, such as moa^74^. This is largely because of an overly coarse understanding of the spatiotemporal dynamics of Polynesian migration across New Zealand. Our high spatiotemporal resolution reconstructions of Māori abundances provide new opportunities to better establish how settlement behaviours of Polynesians impacted the dynamics of past extinctions, pinpointing areas that provided important sanctuaries for biota due to delayed settlement, improving knowledge of faunal collapse and changes in ecological function. If used in combination with proxy archives of paleo-environmental change (charcoal, pollen records etc.), these reconstructions of human colonisation dynamics will provide a more complete understanding of landscape transformation across entire islands at a high spatiotemporal resolution, including for vegetation change and impacts from anthropogenic burning^62^. This is because current maps of landscape transformation for New Zealand are temporally coarse^35^. Moreover, these reconstructions of human colonisation dynamics can provide important information for establishing timing and severity of human-driven changes in the genetic diversity of endemic species, including endangered species, enabling better informed conservation decisions^75^.

Although our modelling shows that Māori are likely to have had little ecological impact on the forests west of the Southern Alps, the pervasive impacts of introduced commensals such as the kiore (*Rattus exulans*) were significant^12^, as was the influence of altered fire regimes in other parts of the archipelago^35^. Accordingly, future modelling exercises that investigate biodiversity change following human colonisation of New Zealand will ideally need to include the likely impacts of commensals and their cascading effects on native, insular biota.

### Broader application

While New Zealand presents a tractable example of human colonisation and expansion, resulting in a globally-significant decline in biodiversity^14^, it is far from unique in this regard. Human arrival and expansion during the Holocene was a major event on many other islands^76^, leading to extinctions, changes in community structure of plants and animals, and wholesale shifts in the structure and function of insular ecosystems^35,76,77^.

Islands across the Pacific Ocean were populated at different times during the Polynesian expansion^13^, often resulting in extreme declines in biodiversity. Among the most heavily impacted islands was Rapa Nui/Easter Island, which lost its entire endemic forest cover following the arrival of Polynesian colonists^78^. Similarly, Polynesians colonized the Hawaiian archipelago in the early 1200s^16^, resulting in a greater loss of native vertebrates (birds) than that following their colonization of New Zealand^77^. Yet each of the Pacific Islands was unique, both in their endemic biodiversity, and in their capacity to support human populations^79^. This surely resulted in different patterns of human population growth and spread across the archipelagos of the Pacific, and different speeds and possibly different mechanisms of biodiversity loss.

In the Indian Ocean, a similar scenario of human colonisation and extinction befell the ratite elephant birds of Madagascar^17^, among other species. The patterns and consequences of human colonisation of Madagascar are even more uncertain than those of New Zealand or Hawai’i, with continuing debates over the latency between human colonisation and extinctions^19,20^, along with the putative driving forces^17^. Likewise, the Caribbean islands lost many endemic vertebrates during the late Holocene (beginning around 6000 BP)^21^, however the spatiotemporal signatures and anthropogenic contribution to these extinctions remains contested^80^.

In each of these cases, the process-explicit modelling approach we used to reconstruct island colonisation of humans across New Zealand could help untangle the potential interdependence between the dynamics of first colonists of an archipelago and the subsequent demographic, geographic and ecological dynamics of its native biota. At a minimum, this would require a dated archaeological record, climate data and ideally either an independent, direct estimate of population size following colonisation (as used here), or one inferred from molecular data. Furthermore, ethical research practices that ensure involvement of Indigenous Peoples, and that archaeological and molecular data with a collective dimension are used appropriately and respectfully are recommended^46^.

## Conclusions

The integration of accurately dated archaeological evidence and spatially explicit population models using a pattern-oriented paradigm enabled reliable and plausible simulations of Māori colonisation and expansion across New Zealand at a fine spatiotemporal resolution. In comparison to commonly used statistical approaches for reconstructing human migration, the modelling protocol we implemented has an advantage in that it can identify the demographic and environmental drivers of rapid colonisation events, including those that took place during periods of climatic stability, producing high resolution projections of abundance patterns that pinpoint migration routes. This is the very information needed to establish how human activities transformed island biodiversity.

Our new approach for reconstructing island colonization by humans has the potential to address outstanding questions concerning the spatiotemporal dynamics of humanity and their ecological impacts on native insular biotas of islands across the Pacific, as well as those of the Caribbean, Mediterranean, Mascarenes and Madagascar. The framework is flexible to future refinements, including the addition of different population growth models, different targets based on new or existing archaeological and paleobiological information, and different simulations of past climate and environmental change.

### Supplementary Information

Supplementary Information.

Supplementary Animation S1.

Supplementary Animation S2.

## Data Availability

All analyses were coded in Program R version 4.0.4 and are described in detail, along with complete data sets, in the Supporting Methods (https://figshare.com/s/02c292e2386633546e2e).
